# Altered Circulating Cytokine Profile Among mRNA‐Vaccinated Young Adults: A Year‐Long Follow‐Up Study

**DOI:** 10.1002/iid3.70194

**Published:** 2025-04-09

**Authors:** Amani Alghamdi, Syed Danish Hussain, Kaiser Wani, Shaun Sabico, Abdullah M. Alnaami, Osama Emam Amer, Nasser M. Al‐Daghri

**Affiliations:** ^1^ Biochemistry Department College of Science King Saud University Riyadh Saudi Arabia

**Keywords:** age, COVID‐19 vaccination, cytokines, gender

## Abstract

**Objectives:**

This longitudinal study aimed to assess the impact of COVID‐19 vaccination on cytokine profile.

**Methods:**

A total of 84 Saudi subjects (57.1% females) with mean age of 27.2 ± 12.3 participated in this longitudinal study. Anthropometric data and fasting blood samples were obtained at baseline and after final vaccination, with an average follow‐up duration of 14.1 ± 3.6 months for adolescents and 13.3 ± 3.0 months for adults, calculated from the first dose of vaccination. Assessment of cytokine profiles was done using commercially available assays.

**Results:**

After follow‐up, a significant increase in weight and body mass index was observed overall (*p* = 0.003 and *p* = 0.002, respectively). Postvaccination, significant increases were observed in several cytokines, including basic fibroblast growth factor 2 (*p* < 0.001), interferon gamma (IFNγ) (*p* = 0.005), interleukin‐1 beta (IL1β) (*p* < 0.001), IL4 (*p* < 0.001), IL6 (*p* = 0.003), IL7 (*p* = 0.001), IL17E (*p* < 0.001), monocyte chemoattractant protein‐1 (MCP1) (*p* = 0.03), MCP3 (*p* = 0.001), tumor necrosis factor alpha (TNFα) (*p* < 0.001), and VEGFA (*p* < 0.001). A significant reduction was observed only in macrophage colony‐stimulating factor (*p* < 0.001). When adjusted for age, epidermal growth factor (EGF), IL4, IL6, MCP3, TNFα, and vascular endothelial growth factor (VEGFA) remained statistically significant. Gender‐based analysis revealed that men experienced greater increases in IL6 (*p* = 0.008), IL4 (*p* = 0.04), and TNFα (*p* = 0.015) compared to women. Age‐based analysis showed that older participants had more pronounced increases in EGF (*p* = 0.011), IL6 (*p* = 0.029), MCP1 (*p* = 0.042), and TNFα (*p* = 0.017), while younger participants had a greater increase in VEGFA (*p* = 0.025).

**Conclusions:**

The findings of this study indicated that COVID‐19 vaccination resulted in an increase in cytokine levels, which signifies the persistence of the humoral immune response to messenger RNA (mRNA) vaccines. This effect may be attributed to the persistent production of spike protein and highly inflammatory nature of mRNA–lipid nanoparticle. Additionally, the results suggested differences in cytokine levels based on gender and age. Notably, the cytokine profile remains favorably altered in young adults who received mRNA vaccinations, even after 1 year.

## 1 Introduction

The COVID‐19 vaccines have demonstrated significant effectiveness in curbing the spread and severity of the virus [1]. Numerous studies have examined the effects of various COVID‐19 vaccines on infection rates, hospitalization rates, and mortality outcomes [1, 2]. The findings consistently demonstrate that COVID‐19 vaccines significantly reduce the incidence of infections, hospitalizations, and deaths [1, 2]. In a study analyzing the impact of vaccination on COVID‐19 outbreaks in the United States, it was reported that vaccines play a critical role in decreasing the incidence of infections, hospitalizations, and mortality, particularly among high‐risk populations [3]. Furthermore, a meta‐analysis assessing survival rates among patients in the United States, stratified by vaccination status, indicated that unvaccinated individuals were 2.46 times more likely to succumb to COVID‐19 compared to their vaccinated counterparts [4].

The COVID‐19 genetic vaccines, including adenoviral‐based vaccines (developed by AstraZeneca and Janssen) and messenger RNA (mRNA) vaccines (developed by Pfizer/BioNTech and Moderna), contain genetic instructions that enable human cells to produce a viral antigen [5]. These vaccines are designed to stimulate the immune system and initiate a protective immune response against the severe acute respiratory syndrome coronavirus‐2 (SARS‐CoV‐2) infection [6]. The specific immune activation mechanisms can vary depending on the type of vaccine used [7]. For instance, monovalent mRNA vaccines, such as the BNT162b2 (Pfizer‐BioNTech) and mRNA‐1273 (Moderna) vaccines, stimulate mRNA to produce spike protein found on the virus' surface. This spike protein then triggers an immune response, leading to the production of antibodies and activation of immune cells [8]. On the other hand, protein subunit vaccines like the NVX‐CoV2373 (Novavax) contain specific viral proteins, that is, the spike protein, which prompt the immune system to recognize and respond to the virus. Some protein subunit vaccines may also include adjuvants to enhance the immune response [8]. The SARS‐CoV‐2 mRNA, encoding the virus's pathogenic spike protein, is encapsulated in lipid nanoparticles and delivered intramuscularly, where it enters host cell cytoplasm to hijack ribosomes for spike protein synthesis via translation [9]. These proteins are displayed on major histocompatibility complexes (MHC‐I) (ubiquitous in nucleated cells) and MHC‐II complexes (specific to antigen‐presenting cells), activating T‐helper (CD4+) cells that release cytokines (e.g., IL‐2 and IL‐4) to stimulate B‐cell differentiation into antibody‐producing plasma cells and memory T‐cell proliferation [9]. T‐cytotoxic (CD8+) cells bind MHC‐I complexes, generating molecules that eliminate future virus‐infected cells while amplifying the broader immune response [9, 10].

COVID‐19 vaccines elicit both the innate and adaptive immune responses [8]. The innate immune response, serving as the initial defense, engages immune cells like macrophages and dendritic cells. These cells identify vaccine components as foreign and trigger an immune reaction. The adaptive immune response generates antibodies and activates T cells that identify and eliminate the virus [8]. The immune system activation stimulated by COVID‐19 vaccines can result in the secretion of cytokines, small proteins that facilitate cell signaling and immune regulation. Epidermal growth factor (EGF) and basic fibroblast growth factor 2 (FGF2) are involved in cellular proliferation and wound healing, contributing to tissue repair postvaccination [11]. In contrast, cytokines such as interferon gamma (IFNγ) and tumor necrosis factor alpha (TNFα) are key players in promoting Th1 immune responses, enhancing the activation of macrophages and cytotoxic T cells [12]. Interleukins, particularly interleukin‐6 (IL6) and IL1β, are associated with inflammation and can influence the severity of immune responses [13]. Meanwhile, IL4 and IL13 are crucial for Th2 responses, which may help in regulating antibody production [14]. Monocyte chemoattractants (MCPs) facilitate the recruitment of immune cells to sites of inflammation [15], while macrophage colony‐stimulating factor (MCSF) supports the survival and proliferation of macrophages [16]. Vascular endothelial growth factor (VEGFA) contributes to angiogenesis, which can aid in the delivery of immune cells to tissues [17]. Recent evidence has demonstrated that cytokines play a crucial role not only in coordinating a sexually‐dimorphic immune response following SARS‐CoV infection [18, 19], but may also serve as predictors of mortality [8, 19, 20].

The specific cytokine profile following vaccination may vary and is still an area of ongoing research [6, 7, 8, 20]. It is imperative to comprehend the immune reaction prompted by these vaccines since early responses to this type of preventive intervention can dictate the strength of both humoral and cellular defensive immunity [21]. While most of the cytokine observations following COVID‐19 vaccinations focused on short durations [8, 20], long‐term follow‐ups are necessary to determine the extent of vaccine's conferred immune protection. To fill this gap, the present longitudinal study aims to explore the changes in cytokine profile among young individuals who have been vaccinated against SARS‐CoV for at least 1 year, specifically those who received mRNA‐type SARS‐CoV2 vaccines.

## 2 Materials and Methods

### 2.1 Participants and Assessment at Prevaccination Visit

This longitudinal study utilized clinical information from a master database collected during an educational intervention program conducted in collaboration with the Saudi Charitable Association of Diabetes. The study population consisted of 84 Saudi adolescents and adults who were recruited during a 5‐month period starting in November 2020, after the lifting of COVID‐19 restrictions but before the initiation of the COVID‐19 vaccination campaign. Participants were followed after receiving their second booster vaccine dose. There were no specific inclusion criteria for participation, but individuals with chronic diseases were excluded from the study. Sociodemographic and anthropometric data were collected during the initial screening and after the second booster dose. Fasting blood samples were obtained after an 8‐h fasting period at both time points. Anthropometric measurements included height (in centimeters), weight (in kilograms), body mass index (BMI, calculated as kilograms per square meter), and waist and hip circumferences (in centimeters). Blood pressure (systolic and diastolic, in millimeters of mercury) was measured by trained nurses using standard procedures, and the average of two readings was recorded. The study was approved by the Institutional Review Board (IRB) of the College of Medicine, King Saud University (KSU), Riyadh, Saudi Arabia (no. E‐23‐7494).

### 2.2 Postvaccination Assessment

The postvaccination recruitment phase commenced in November 2021, with an average follow‐up duration of 14.1 ± 3.6 months for adolescents and 13.3 ± 3.0 months for adults. During the follow‐up visit, participants underwent the routine procedures of an 8‐h fasting blood sample collection and anthropometric assessments. Additionally, they were asked a series of questions concerning their COVID‐19 vaccination history, including details about the types of first, second, and booster doses they received and the dates of vaccination. Participants were also queried about whether they had contracted a COVID‐19 infection and, if so, the specific timeframe during the study period in which it occurred. To ensure accuracy, the provided information on vaccination dates, vaccine types, and COVID‐19 infections was cross‐verified with the vaccination and infection records maintained by the Ministry of Health (MOH) in the Kingdom of Saudi Arabia (KSA).

### 2.3 Assessment of Cytokines

A total of 18 serum cytokines including EGF, FGF2, IFNγ, interleukin‐1 alpha (IL1α), IL1β, IL4, IL6, IL7, IL13, IL17E, IL17F, monocyte chemoattractant protein‐1 (MCP1), MCP3, MCSF, platelet‐derived growth factor (PDGFAA), transforming growth factor alpha (TGFα), TNFα, and VEGFA were assessed using multiplex assay kits, specifically the Milliplex human high‐sensitivity T cell magnetic bead panel. These kits rely on the Luminex xMAP Technology platform developed by Luminex Corporation (Austin, TX, USA).

### 2.4 Statistical Analysis

Data were analyzed using SPSS (version 22, Chicago, IL, USA). Continuous data were presented as mean ± standard deviation (SD) for normal variables and non‐normal variables were presented as median (25th and 75th) percentiles. Categorical data were presented as frequencies. Paired sample *t*‐test and paired samples Wilcoxon test were used to assess differences between baseline and follow‐up data for normal and non‐normal data, respectively. Group differences were assessed by analyzing change in pre–post data using independent sample *t*‐test and Mann–Whitney *U* test for normal and non‐normal variables, respectively. The R package, Gplot, was used to draw the heatmaps. *p*‐value < 0.05 was considered statistically significant.

## 3 Results

A total of 84 subjects participated in this study with the mean age of 27.2 ± 12.3 years with 36 males and 48 females. Postvaccination significant change in weight was observed that increased from 66.6 ± 17.2 to 70.3 ± 16.7 (*p* = 0.003) along with BMI that increased from 25.4 ± 5.8 to 26.9 ± 5.6 (*p* = 0.002).

Table 1 shows inflammatory markers at baseline and follow‐up. Postvaccination significant increase in several markers were observed including FGF2 from 43.5 (17.1–179.0) to 46.2 (18.9–217.8) (*p* < 0.001), IFNγ from 3.5 (1.8–12.9) to 3.5 (2.3–17.0) (*p* = 0.005), IL1β from 17.2 (9.6–24.2) to 22.8 (14.3–31.1) (*p* < 0.000), IL4 from 7.3 (4.1–12.5) to 9.5 (6.2–15.1), (*p* < 0.001), IL6 from 4.0 (1.1–10.2) to 6.2 (1.6–11.3), (*p* = 0.003), IL7 from 10.9 (5.7– 15.2) to 12.5 (8.4–19.9) (*p* = 0.001), IL17E from 408.9 (218.7–621.8) to 544.4 (273.0–772.9) (*p* < 0.001), MCP1 from 372.7 (262.1–584.4) to 421.4 (337.7–730.9) (*p* = 0.03), MCP3 from 39.7 (23.8–63.6) to 45.3 (29.7–70.8) (*p* = 0.001), TNFα from 18.5 (4.9–65.3) to 22.6 (13.0–83.9) (*p* < 0.001), and VEGFA from 112.8 (46.0–212.7) to 255.3 (151.4–383.1) (*p* < 0.001). Postvaccination reduction was only observed in MCSF which decreased from 744.1 (226.5–1379.2) to 393.2 (154.4–794.0) (*p* < 0.001). Additionally, when analysis was adjusted for age only EGF, IL4, IL6, MCP3, TNFα, and VEGFA remained statistically significant.

**Table 1:** Descriptive statistics of pre and postvaccination.

Parameters	Baseline	Follow‐up	*p*‐Value	*p*‐Value*
*N*	84			
Age	27.2 ± 12.3			
M/F	36/48		0.19	
Weight (kg)	66.6 ± 17.2	70.3 ± 16.7	0.003	< 0.001
Waist (cm)	79.8 ± 13.6	80.7 ± 14.1	0.46	0.03
Hips (cm)	97.6 ± 15.4	96.5 ± 15.4	0.49	0.001
BMI (kg/m^2^)	25.4 ± 5.8	26.9 ± 5.6	0.002	< 0.001
WHR	0.8 ± 0.1	0.8 ± 0.1	0.10	0.18
Systolic BP (mmHg)	118.5 ± 12.9	115.9 ± 11.8	0.11	0.020
Diastolic BP (mmHg)	72.7 ± 11.1	72.0 ± 7.2	0.54	0.19
EGF (pg/mL)	574.3 (290.0–793.7)	479.7 (248.3–930.0)	0.92	0.001
FGF2 (pg/mL)	43.5 (17.1–179.0)	46.2 (18.9–217.8)	0.001	0.23
IFNγ (pg/mL)	3.5 (1.8–12.9)	3.5 (2.3–17.0)	0.005	0.70
IL1α (pg/mL)	84.6 (39.0–130.0)	88.4 (42.1–133.5)	0.20	0.34
IL1β (pg/mL)	17.2 (9.6–24.2)	22.8 (14.3–31.1)	< 0.001	0.11
IL4 (pg/mL)	7.3 (4.1–12.5)	9.5 (6.2–15.1)	< 0.001	0.03
IL6 (pg/mL)	4.0 (1.1–10.2)	6.2 (1.6–11.3)	0.003	0.014
IL7 (pg/mL)	10.9 (5.7–15.2)	12.5 (8.4–19.9)	< 0.001	0.46
IL13 (pg/mL)	46.9 (28.1–69.7)	47.9 (30.1–73.5)	0.46	0.31
IL17E (pg/mL)	408.9 (218.7–621.8)	544.4 (273.0–772.9)	< 0.001	0.095
IL17F (pg/mL)	22.5 (12.7–183.9)	22.7 (13.8–203.0)	0.13	0.63
MCP1 (pg/mL)	372.7 (262.1–584.4)	421.4 (337.7–730.9)	0.033	0.57
MCP3 (pg/mL)	39.7 (23.8–63.6)	45.3 (29.7–70.8)	0.001	0.006
MCSF (pg/mL)	744.1 (226.5–1379.2)	393.2 (154.4–794.0)	< 0.001	0.15
PDGFAA (pg/mL)	10,414.0 (6677.8–14,206.8)	9962.2 (5932.7–13,233.8)	0.22	0.28
TGFα (pg/mL)	16.3 (7.2–25.4)	17.0 (8.4–25.1)	0.39	0.44
TNFα (pg/mL)	18.5 (4.9–65.3)	22.6 (13.0–83.9)	< 0.001	0.001
VEGFA (pg/mL)	112.8 (46.0–212.7)	255.3 (151.4–383.1)	< 0.001	< 0.001

*Note:* Data presented as mean ± SD for normal variables whereas median (Q1–Q3) for non‐normal variables; *p* < 0.05 considered significant.

Abbreviation: WHR, waist to hip ratio.

* Indicates *p*‐values adjusted for age.

**Figure 1:**
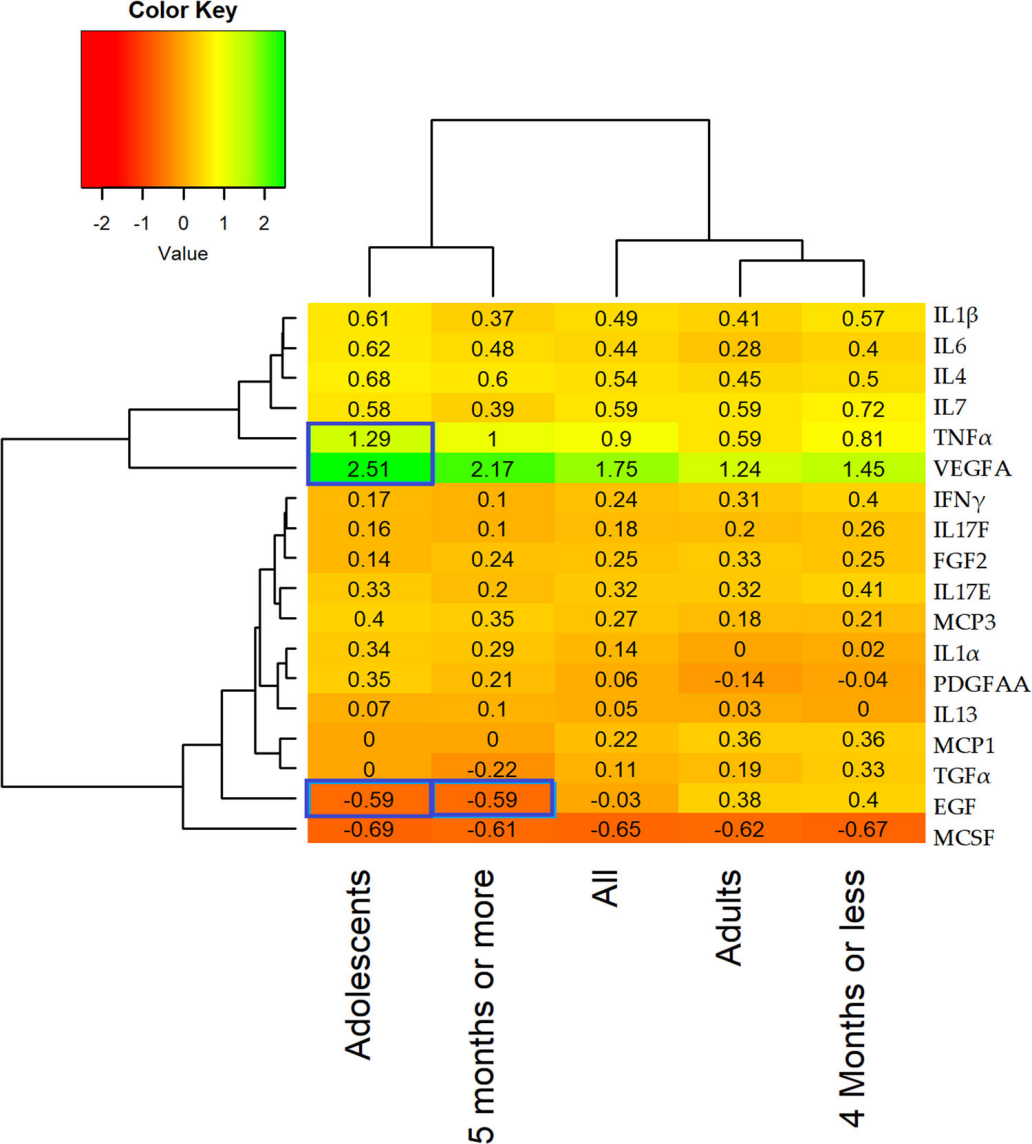
Log2 fold change in biomarkers postvaccination among select groups.

Figure 1 shows log2 fold changes in biomarkers postvaccination. The changes inside the blue boxes show significant log2 fold change between counterpart category. The highest log2 fold increase after vaccination was observed in VEGFA while the lowest was observed in MCSF. The responses of biomarkers continued to be higher as compared to baseline level for most of the cytokines with the exception of MCSF and EGF as indicated by log2 fold change in Figure 1. MCSF levels declined in all the subcategories whereas, EGF declined only in adolescent and those who receive their last dose 5 months ago or earlier. Significance testing revealed that reduction in EGF in adolescents and those who receive their last dose 5 months ago or earlier were significantly lower as compared to adults and those who took their last dose 4 months ago or earlier, respectively, and witnessed increase in EGF. Furthermore, increase in log2 fold change in VEGFA and TNFα was also significantly higher as compared to log2 fold change observed in adults.

**Figure 2:**
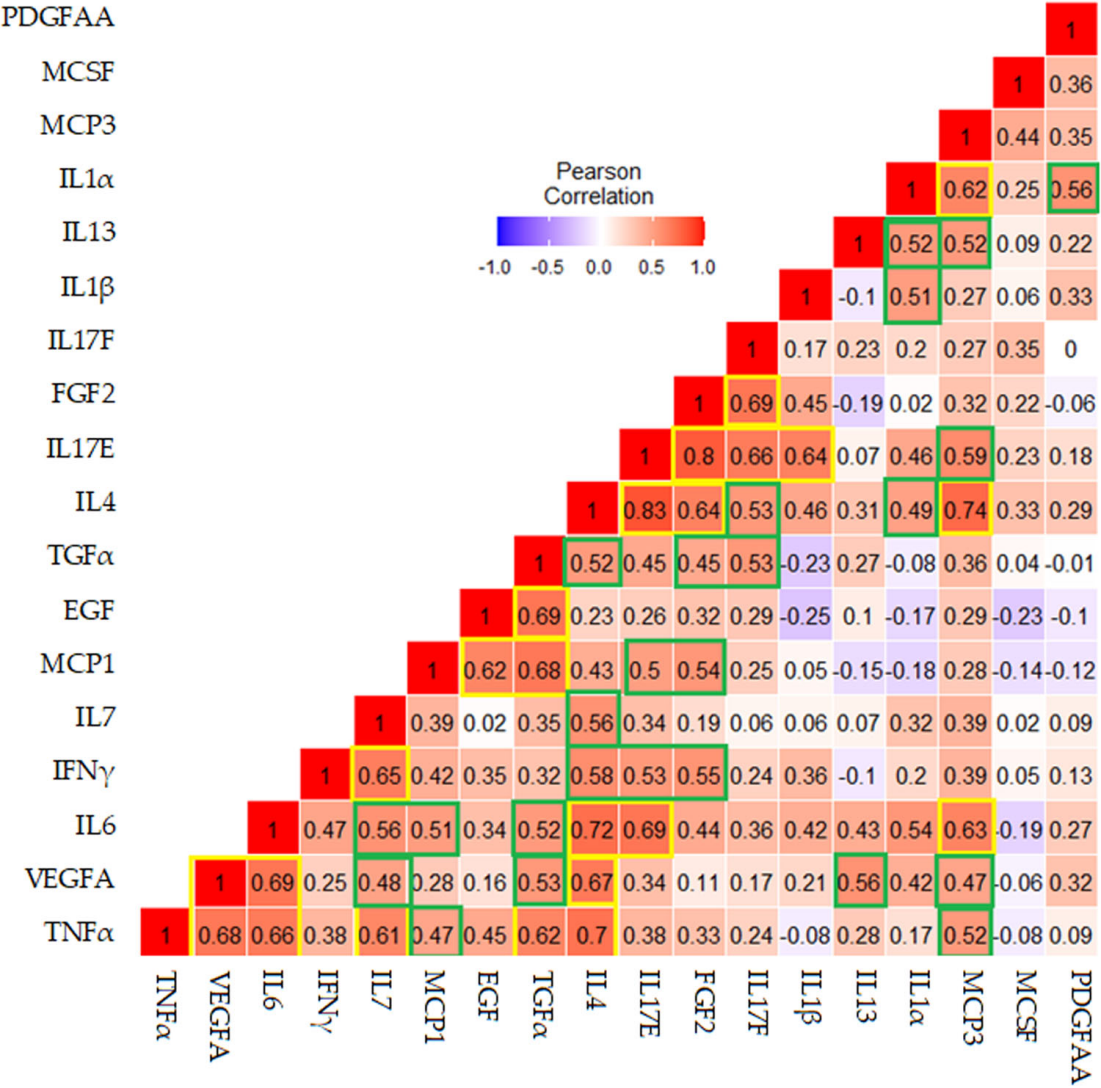
Correlation between log2 fold change postvaccination among serum cytokines.

Figure 2 shows correlations between log2 fold change postvaccination among serum cytokines. Pairwise correlations were computed using the Pearson correlation coefficient for the log2 fold changes of 18 biomarkers postvaccination. Darker red color denotes a strong positive relationship whereas blue color shows negative relationship between cytokines. The yellow boxes show strong to moderate positive correlations with the significance level of < 0.001 and identify the cluster of associations featuring TNFα, VEGFA, IL6, IL7, TFGα, IL4, MCP1, EGF, IL17E, FGF2, IL17F, IL1β, and MCP3. Furthermore, green boxes show moderate to weak positive correlations with the significance level of *p* < 0.05 including cluster of associations featuring IL7, MCP1, TGFα, IL13, MCP3, IFNγ, IL4, IL17E, IL1β, and IL1α.

Table 2 shows inflammatory markers at baseline and follow‐up according to gender. Several inflammatory markers changed significantly across male and female participants. Since female participants were significantly older than male participants, therefore age‐adjusted differences were obtained to identify differences between the genders. In comparison to women, men experienced more significant increase in IL6 with median change of 1.1 (0.1–7.0) as compared to median change of 0.4 (−1.4 to 2.4) in women (*p* = 0.008). Furthermore, IL‐4 concentration was also found to be significantly elevated in men with median concentration of 1.9 (0.3–3.1) as compared to women with median concentration of 1.5 (0.1–4.4) (*p* = 0.04). Similarly, postvaccination changes in TNFα was significantly higher in male participants with median change of 10.3 (0.0–15.8) as compared to female participants with median change of 7.1 (0.0–27.1) (*p* = 0.015).

**Table 2:** Descriptive statistics of pre and postvaccination according to gender.

Parameters	Female (*N* = 48)				Male (*N* = 36)				Group effect	
	Prevaccination	Postvaccination	Change	*p*‐Value	Prevaccination	Postvaccination	Change	*p*‐Value	*p*‐Value	*p*‐Value*
Age	30.7 ± 13.0				22.6 ± 9.9				0.002	—
Weight (kg)	65.1 ± 17.4	66.9 ± 15.6	1.8 ± 9.1	0.18	68.7 ± 17.0	75.0 ± 17.1	6.3 ± 12.9	0.006	0.003	0.96
Waist (cm)	78.0 ± 12.5	76.7 ± 13.7	−1.2 ± 11.1	0.45	82.3 ± 14.8	86.0 ± 12.9	3.8 ± 10.6	0.04	0.024	0.10
Hips (cm)	101.9 ± 14.9	96.2 ± 16.7	−5.7 ± 14.1	0.007	91.9 ± 14.4	97.0 ± 13.8	5.1 ± 11.8	0.014	0.001	0.007
BMI (kg/m^2^)	26.1 ± 6.3	26.9 ± 5.6	0.8 ± 4.0	0.17	24.6 ± 5.0	27.0 ± 5.6	2.4 ± 4.5	0.003	0.003	0.96
WHR	0.8 ± 0.1	0.8 ± 0.1	0.0 ± 0.1	0.06	0.9 ± 0.1	0.9 ± 0.1	0.0 ± 0.1	0.90	0.048	0.38
Systolic BP (mmHg)	115.6 ± 12.3	112.5 ± 11.3	−3.1 ± 16.1	0.18	122.4 ± 12.8	120.5 ± 10.9	−1.9 ± 13.8	0.40	0.87	0.32
Diastolic BP (mmHg)	77.0 ± 10.8	72.9 ± 5.6	−4.1 ± 10.6	0.011	67.1 ± 8.9	70.7 ± 8.8	3.6 ± 11.3	0.06	0.006	0.008
EGF (pg/mL)	490.3 (167.6–823.9)	475.3 (267.2–889.0)	0.0 (−297.0 – 418.9)	0.76	659.6 (508.4–793.7)	491.2 (200.5–1146.7)	−5.6 (−395.3 to 363.3)	0.76	0.24	0.41
FGF2 (pg/mL)	113.4 (21.5–188.9)	127.4 (25.7–236.6)	8.5 (−0.9 to 31.4)	0.005	20.7 (11.2–141.6)	24.9 (13.3–188.9)	2.0 (−2.2 to 13.0)	0.09	0.92	0.54
IFNγ (pg/mL)	4.9 (2.4–14.1)	5.3 (2.4–18.1)	0.7 (−0.5 to 3.6)	0.02	2.3 (1.4–10.8)	2.8 (1.9–12.4)	0.2 (0.0–1.6)	0.11	0.77	0.19
IL1α (pg/mL)	99.2 (47.0–130.0)	94.7 (57.0–135.7)	4.3 (−7.4 to 33.2)	0.04	47.0 (33.4–126.5)	58.2 (33.4–119.2)	0.0 (‐−7.6 to 12.3)	0.50	0.21	0.47
IL1β (pg/mL)	18.7 (10.0–26.3)	27.0 (14.5–34.2)	5.2 (−0.4 to 10.6)	0.000	14.5 (7.0–22.7)	19.2 (11.8–26.2)	0.8 (−0.9 to 7.6)	0.034	0.53	0.14
IL4 (pg/mL)	10.0 (5.2–12.8)	13.2 (6.4–15.3)	1.5 (0.1–4.4)	0.000	5.1 (3.1–10.3)	7.3 (5.4–13.4)	1.9 (0.3–3.1)	< 0.001	0.08	0.040
IL6 (pg/mL)	5.4 (1.9–10.8)	5.9 (1.6–12.1)	0.4 (−1.4 to 2.4)	0.30	2.0 (0.5–7.6)	6.6 (1.6–11.0)	1.1 (0.1–7.0)	0.001	0.000	0.008
IL7 (pg/mL)	11.3 (8.5–15.7)	13.2 (9.1–23.2)	3.5 (−0.7 to 8.3)	0.001	5.5 (3.1–13.0)	12.1 (5.6–17.7)	3.5 (0.0–6.9)	0.015	0.12	0.92
IL13 (pg/mL)	61.6 (42.0–74.4)	60.2 (32.3– 81.4)	1.2 (−11.2 to 15.1)	0.53	32.4 (25.2–53.6)	37.0 (29.6–57.6)	5.4 (−14.8 to 14.2)	0.72	0.35	0.68
IL17E (pg/mL)	485.7 (295.0–672.8)	611.3 (295.0–836.0)	75.2 (−59.2 to 309.5)	0.004	269.8 (179.5–565.6)	453.4 (257.2–639.3)	33.3 (0.0–117.8)	0.016	0.97	0.80
IL17F (pg/mL)	34.3 (16.8–210.8)	44.2 (15.7–272.4)	4.8 (−6.0 to 33.7)	0.10	16.8 (9.8–58.1)	17.5 (12.4–67.3)	0.0 (−1.4 to 3.5)	0.75	0.42	0.20
MCP1 (pg/mL)	360.3 (252.4–564.2)	437.8 (348.4– 805.7)	79.5 (−72.5 to 204.6)	0.01	378.9 (266.5–602.9)	415.1 (263.5–590.7)	0.0 (−126.8 to 149.3)	0.72	0.07	0.09
MCP3 (pg/mL)	43.8 (27.6–63.6)	50.2 (33.5–77.0)	6.5 (0.0–17.0)	0.001	29.7 (20.8–57.4)	33.1 (28.7–62.0)	4.1 (−1.6 to 10.4)	0.12	0.86	0.48
MCSF (pg/mL)	898.1 (121.3–1278.8)	527.0 (140.5–762.8)	−107.8 (−668.6 to 29.4)	0.001	465.2 (320.7–1641.0)	306.4 (213.6–847.0)	−120.2 (−433.4 to 0.0)	0.001	0.57	0.10
PDGFAA (pg/mL)	9709.4 (7002.6–13,932.5)	9545.2 (5475.1–13,181.2)	−1568.8 (−4359.9 to 1551.9)	0.17	11180.9 (6358.2–14,775.1)	11294.6 (7998.2–13,238.4)	0.0 (−2583.3 to 1630.7)	0.92	0.26	0.84
TGFα (pg/mL)	18.3 (10.9–26.5)	22.7 (11.3–29.1)	0.3 (−4.9 to 9.9)	0.31	9.7 (4.1–18.2)	11.1 (4.6–18.3)	0.0 (−2.0 to 3.0)	0.89	0.38	0.29
TNFα (pg/mL)	26.7 (10.9–67.4)	47.1 (14.5–94.8)	7.1 (0.0–27.1)	0.003	12.4 (2.3–54.3)	18.5 (13.0–70.6)	10.3 (0.0–15.8)	0.001	0.04	0.015
VEGFA (pg/mL)	159.0 (91.4–235.8)	260.5 (170.2–400.1)	98.6 (0.0–206.2)	< 0.001	54.2 (25.6–130.0)	250.1 (138.0–366.0)	133.0 (51.5–291.1)	0.000	0.001	0.62

*Note:* Data presented as mean ± SD for normal variables whereas median (Q1–Q3) for non‐normal variables; *p* < 0.05 considered significant.

Abbreviation: WHR, waist to hip ratio.

* Indicates age‐adjusted *p*‐values.

Table 3 shows inflammatory markers at baseline and follow‐up according to age groups. In comparison to younger participants, older adults experienced more pronounced increase in EGF with median change of 54.3 (−293.5 to 445.6) as compared to younger participants who experienced decrease in EGF with median change of −137.4 (−586.2 to 60.7) (*p* = 0.011). Similarly, postvaccination change in IL6 was significantly higher in older participants with median change of 1.2 (−0.8 to 3.3) as compared to younger participants with median change of 0.7 (0.1–3.0) (*p* = 0.029). Furthermore, older participants also reported higher median change of 84.7 (−12.9 to 201.5) in MCP1 as compared to younger participants who experienced decrease in MCP1 with median change of −22.5 (−86.4 to 116.2) (*p* = 0.042). In addition, TNFα was also increased significantly in older participants with median change of 15.8 (0.0–31.8) as compared to younger participants who experienced median increase of 6.5 (0.9–11.1) (*p* = 0.017). However, VEGFA increased significantly in younger participants with median change of 133.0 (32.4–234.7) as compared to median change of 98.2 (0.0–215.2) in older participants (*p* = 0.025).

**Table 3:** Descriptive statistics of pre and postvaccination according to age group.

Parameters	Adolescents (*N* = 35)				Adults (*N* = 49)				Group effect
	Prevaccination	Postvaccination	Change	*p*‐Value	Prevaccination	Postvaccination	Change	*p*‐Value	*p*‐Value
Age	15.4 ± 1.1				35.6 ± 9.4				< 0.001
Weight (kg)	57.4 ± 16.0	67.0 ± 17.7	9.6 ± 10.8	< 0.001	73.2 ± 15.0	72.7 ± 15.7	−0.5 ± 9.2	0.72	< 0.001
Waist (cm)	75.4 ± 13.3	80.4 ± 13.2	5.0 ± 9.4	0.004	83.0 ± 13.1	81.0 ± 14.8	−2.0 ± 11.4	0.23	0.001
Hips (cm)	89.0 ± 13.8	96.2 ± 13.7	7.3 ± 9.6	< 0.001	103.8 ± 13.5	96.7 ± 16.6	−7.0 ± 13.9	0.001	< 0.001
BMI (kg/m^2^)	22.2 ± 5.3	26.1 ± 5.9	3.8 ± 4.6	< 0.001	27.7 ± 5.0	27.5 ± 5.3	−0.2 ± 3.2	0.69	< 0.001
WHR	0.8 ± 0.1	0.8 ± 0.1	0.0 ± 0.1	0.61	0.8 ± 0.1	0.8 ± 0.1	0.0 ± 0.1	0.02	0.006
Systolic BP (mmHg)	118.5 ± 15.4	121.3 ± 12.5	2.8 ± 14.5	0.26	118.6 ± 10.9	112.0 ± 9.6	−6.5 ± 14.4	0.003	0.004
Diastolic BP (mmHg)	71.2 ± 10.4	72.0 ± 9.4	0.8 ± 11.9	0.69	73.8 ± 11.6	71.9 ± 5.2	−1.9 ± 11.2	0.24	0.61
EGF (pg/mL)	590.9 (254.3–793.7)	327.2 (132.7–555.5)	−137.4 (−586 to 60.7)	0.09	528.8 (310.2–801.9)	650.3 (345.8 – 1085.3)	54.3 (−293.5 to 445.6)	0.11	0.01
FGF2 (pg/mL)	18.9 (13.3–27.3)	24.4 (13.3–38.6)	2.0 (−1.8 to 8.2)	0.12	172.9 (141.6–215.3)	209.9 (141.6 – 240.2)	23.2 (0.0–55.4)	0.003	0.67
IFNγ (pg/mL)	1.9 (1.4–2.6)	2.4 (1.3–3.2)	0.2 (−0.7 to 1.1)	0.38	12.9 (4.6–17.0)	17.0 (4.3 – 21.1)	1.3 (0.0–5.2)	0.01	0.44
IL1α (pg/mL)	41.4 (32.8–48.3)	46.4 (33.4–58.2)	5.2 (−5.6 to 19.1)	0.11	121.7 (92.1–148.7)	116.7 (88.4 – 157.0)	0.0 (−13.6 to 33.2)	0.59	0.27
IL1β (pg/mL)	11.0 (6.5–17.7)	15.4 (10.5–23.0)	2.8 (−1.2 to 7.0)	0.01	21.9 (12.9–29.4)	28.3 (20.1 – 36.1)	4.5 (0.0–10.4)	< 0.001	0.75
IL4 (pg/mL)	4.2 (3.1–5.2)	6.3 (5.1–7.5)	1.8 (0.6–2.4)	< 0.001	11.0 (9.5–13.9)	14.4 (11.9 – 15.4)	1.5 (0.0–5.2)	< 0.001	0.16
IL6 (pg/mL)	1.1 (0.5–2.2)	1.6 (1.2–4.8)	0.7 (0.1–3.0)	0.01	8.9 (5.4–12.6)	10.2 (5.4 – 15.4)	1.2 (−0.8 to 3.3)	0.05	0.03
IL7 (pg/mL)	5.5 (3.3–13.0)	8.0 (5.2–12.1)	2.8 (−1.3 to 4.7)	0.09	11.7 (9.1–16.5)	17.7 (11.9–24.4)	5.1 (0.0–9.3)	< 0.001	0.99
IL13 (pg/mL)	34.9 (25.2–45.9)	35.9 (25.2–45.9)	4.6 (0.0–12.6)	0.37	61.6 (39.9–78.1)	65.4 (37.0–93.1)	0.0 (−15.5 to 18.7)	0.70	0.53
IL17E (pg/mL)	241.2 (166.2–344.7)	282.5 (205.7–429.9)	33.3 (−13.1 to 117.8)	0.08	578.6 (382.5–694.1)	702.1 (523.9–861.6)	92.7 (0.0–309.5)	0.002	0.73
IL17F (pg/mL)	14.4 (9.8–21.8)	15.5 (12.1–21.0)	0.0 (−1.7 to 6.2)	0.29	183.9 (27.2–258.0)	198.7 (27.2–276.2)	3.6 (−14.7 to 35.6)	0.27	0.88
MCP1 (pg/mL)	549.5 (291.0–730.4)	413.1 (351.3–590.7)	−22.5 (−86.4 to 116.2)	0.77	343.5 (249.5–468.3)	427.0 (333.5–762.9)	84.7 (−12.9 to 201.5)	0.005	0.04
MCP3 (pg/mL)	26.0 (18.2–34.9)	30.7 (25.5–38.9)	5.8 (0.0–10.2)	0.003	61.4 (42.2–68.3)	66.5 (48.5–80.0)	6.5 (−4.8 to 18.7)	0.02	0.24
MCSF (pg/mL)	327.4 (114.4–435.4)	175.3 (112.0–301.0)	−95.5 (−268.7 to 23.9)	0.02	1172.0 (505.9–1496.8)	684.3 (393.2–847.0)	−347.1 (−662.2 to 0.0)	< 0.001	0.58
PDGFAA (pg/mL)	8583.5 (6358–11,181)	9472.4 (5932.7–10,883.9)	149.4 (−2578.4 to 1540.3)	0.94	11731.7 (7970.7–17,250.8)	11120.0 (6401.0–14,694.0)	−1519.9 (−4913.1 to 1627.5)	0.21	0.15
TGFα (pg/mL)	6.9 (3.9–9.8)	7.5 (4.4–11.1)	0.2 (−3.1 to 5.3)	0.66	22.0 (16.5–27.1)	23.8 (16.8– 29.6)	0.0 (−4.5 to 9.3)	0.44	0.78
TNFα (pg/mL)	6.9 (2.7–14.5)	12.8 (9.0–19.5)	6.5 (0.9−11.1)	0.001	59.9 (26.7–75.9)	78.5 (26.7–96.4)	15.8 (0.0–31.8)	0.005	0.02
VEGFA (pg/mL)	60.7 (12.5–159.0)	231.2 (139.4–400.1)	133.0 (32.4–234.7)	< 0.001	153.4 (65.3–235.8)	281.5 (200.3– 366.0)	98.2 (0.0–215.2)	< 0.001	0.02

*Note:* Data presented as mean ± SD for normal variables whereas median (Q1–Q3) for non‐normal variables; *p* < 0.05 considered significant.

Abbreviation: WHR, waist to hip ratio.

Table 4 shows inflammatory markers at baseline and follow‐up according to time to last dose. Participants who got the last dose of Covid vaccine 4 months ago or less reported higher increase in EGF with median change of 85.7 (−297.0 to 438.4) as compared to participants who got their last dose of Covid vaccine 5 months ago or more with median change of −130.2 (−447.8 to 0.0) (*p* = 0.009). Similarly, median change of 6.5 (0.8–12.3) in IL1β was also significantly higher in participants who got the last dose 4 months ago as compared to median change of 0.0 (−1.3 to 4.3) in participants who got their last dose 5 months or more (*p* = 0.04). Median change of 95.1 (−25.7 to 259.7) in MCP1 was also higher in those who got their last dose 4 months ago or earlier as compared to median change of −6.5 (−83.8 to 84.1) who got their last dose of vaccine 5 or more months ago (*p* = 0.02). After further examination, it was found that the group whose last vaccine dose was more than 5 months ago was notably younger than the other group. Upon adjusting for age differences, the analysis showed that disparities in EGF, IL1β, and MCP1 were no longer statistically significant.

**Table 4:** Descriptive statistics of pre and postvaccination according to time to last dose.

Parameters	4 Months or less (*N* = 48)				5 Months or more (*N* = 36)				Group effect	
	Prevaccination	Postvaccination	Change	*p*‐Value	Prevaccination	Postvaccination	Change	*p*‐Value	*p*‐Value	*p*‐Value*
Age	33.8 ± 11.1				18.4 ± 7.6				< 0.001	—
Weight (kg)	70.2 ± 16.0	72.2 ± 15.1	2.0 ± 7.9	0.08	61.8 ± 17.9	67.9 ± 18.5	6.0 ± 14.0	0.014	0.045	0.84
Waist (cm)	82.4 ± 13.7	81.0 ± 14.9	−1.3 ± 12.0	0.45	76.4 ± 13.0	80.3 ± 13.1	3.9 ± 9.2	0.015	0.026	0.26
Hips (cm)	102.1 ± 15.1	96.0 ± 17.0	−6.1 ± 15.1	0.008	91.6 ± 13.8	97.2 ± 13.3	5.6 ± 9.4	0.001	< 0.001	0.08
BMI (kg/m^2^)	26.8 ± 5.5	27.5 ± 5.1	0.8 ± 2.9	0.07	23.7 ± 5.8	26.1 ± 6.1	2.4 ± 5.6	0.013	0.045	0.85
WHR	0.8 ± 0.1	0.9 ± 0.1	0.0 ± 0.1	0.033	0.8 ± 0.1	0.8 ± 0.1	0.0 ± 0.1	0.48	0.09	0.34
Systolic BP (mmHg)	119.5 ± 10.5	112.9 ± 10.6	−6.7 ± 13.9	0.002	117.3 ± 15.5	120.0 ± 12.2	2.8 ± 15.2	0.28	0.011	0.33
Diastolic BP (mmHg)	74.2 ± 12.1	71.2 ± 4.5	−3.0 ± 11.0	0.06	70.7 ± 9.5	72.9 ± 9.7	2.2 ± 11.6	0.26	0.14	0.36
EGF (pg/mL)	518.2 (153.5– 810.9)	537.8 (248.3–1023.9)	85.7 (−297.0 to 438.4)	0.15	623.7 (454.6–739.0)	434.8 (232.4–674.9)	−130.2 (−447.8 to 0.0)	0.08	0.009	0.37
FGF2 (pg/mL)	165.5 (27.3– 209.9)	197.0 (27.3–240.2)	23.2 (−1.8 to 67.4)	0.001	20.6 (13.3–42.1)	24.4 (15.2–47.6)	1.5 (−0.9 − 8.9)	0.17	0.59	0.76
IFNγ (pg/mL)	11.4 (3.6–14.3)	15.0 (2.8–21.1)	1.3 (0.0–5.2)	0.004	2.1 (1.6–3.3)	2.8 (1.5–4.3)	0.3 (−0.5 to 1.1)	0.24	0.56	0.63
IL1α (pg/mL)	116.1 (70.7–145.0)	109.4 (68.2–153.4)	2.5 (−12.4–33.6)	0.23	47.0 (33.4–73.3)	57.0 (33.4–78.3)	0.0 (−7.4 to 19.1)	0.47	0.77	0.31
IL1β (pg/mL)	21.4 (12.9–26.3)	28.3 (21.0–36.1)	6.5 (0.8–12.3)	< 0.001	11.8 (6.5–18.9)	16.2 (10.5–22.8)	0.0 (−1.3 to 4.3)	0.24	0.035	0.58
IL4 (pg/mL)	10.7 (5.1–13.7)	13.9 (9.4–15.5)	2.0 (0.4–5.3)	< 0.001	5.2 (3.4–5.9)	6.4 (5.1–8.1)	1.6 (0.0–2.8)	< 0.001	0.96	0.56
IL6 (pg/mL)	7.8 (3.3–11.6)	9.2 (5.3–14.8)	1.5 (−0.5 to 6.0)	0.013	1.2 (0.5–3.9)	1.6 (1.1–5.4)	0.5 (0.0–1.0)	0.09	0.31	0.60
IL7 (pg/mL)	11.4 (8.1–16.1)	17.7 (12.4–24.4)	6.2 (1.5–9.5)	< 0.001	9.0 (3.7–13.4)	8.7 (5.4–12.0)	0.8 (−2.4 to 4.3)	0.40	0.08	0.77
IL13 (pg/mL)	61.6 (45.9–73.7)	65.4 (38.0–93.1)	0.0 (−11.6 to 18.7)	0.47	35.3 (20.6–51.6)	35.9 (25.2–60.8)	4.6 (−12.1 to 12.6)	0.54	0.44	0.77
IL17E (pg/mL)	562.9 (382.5– 683.4)	672.4 (450.9–853.1)	104.8 (0.0–288.7)	0.001	257.2 (172.9–387.5)	282.5 (218.7–525.0)	22.9 (−13.0 to 104.1)	0.14	0.45	0.63
IL17F (pg/mL)	159.3 (20.2–214.4)	191.8 (19.1–272.4)	3.6 (−5.6 to 35.6)	0.12	16.4 (11.3–29.9)	17.9 (12.4–27.2)	0.0 (−1.6 to 5.7)	0.55	0.60	0.96
MCP1 (pg/mL)	343.5 (252.6–553.8)	427.0 (349.1–802.2)	95.1 (−25.7 to 259.7)	0.008	454.7 (285.2–626.9)	395.5 (288.1–582.3)	−6.5 (−83.8 to 84.1)	0.63	0.020	0.12
MCP3 (pg/mL)	58.9 (42.2–64.6)	65.6 (37.1–80.0)	6.6 (−1.2 to 19.9)	0.006	26.9 (19.5–37.3)	31.6 (26.6–44.9)	4.6 (0.0 − 10.0)	0.029	0.66	0.74
MCSF (pg/mL)	1172.0 (397.7–1496.8)	664.0 (301.0–847.0)	−297.8 (−662.2 to 0.0)	0.000	305.6 (111.6–475.4)	198.3 (104.0– 333.2)	−79.3 (−306.1 to 27.7)	0.018	0.74	0.31
PDGFAA (pg/mL)	11637.6 (6535.9–17,250.8)	10883.9 (5542.7– 14,150.4)	−1499.9 (−4913.1 to 1834.9)	0.39	9015.2 (7096.4–11,376.7)	9545.2 (7402.2–11,235.8)	−35.5 (−2386.1 to 1112.0)	0.43	0.36	0.39
TGFα (pg/mL)	20.5 (14.5–25.9)	22.9 (16.5–29.9)	1.0 (−2.4 to 10.5)	0.07	9.7 (4.4–16.7)	9.7 (5.0–16.1)	−0.8 (−5.7 to 4.5)	0.29	0.052	0.23
TNFα (pg/mL)	53.2 (17.4–69.7)	74.7 (18.6–94.8)	15.8 (0.0–33.9)	0.001	10.5 (3.2–18.5)	15.1 (7.9–22.6)	6.2 (0.0 − 12.0)	0.004	0.62	0.50
VEGFA (pg/mL)	109.3 (55.5–208.8)	261.9 (166.5–347.8)	110.1 (29.0–220.5)	< 0.001	127.5 (28.8–271.4)	237.9 (143.2–411.2)	116.3 (2.5–262.9)	0.000	0.55	0.39

*Note:* Data presented as mean ± SD for normal variables whereas median (Q1–Q3) for non‐normal variables; *p* < 0.05 considered significant.

Abbreviation: WHR, waist to hip ratio.

* Indicates *p*‐value adjusted for age.

## 4 Discussion

The results of this study demonstrated that COVID‐19 vaccination resulted in an increase in cytokine levels, signifying continued immune system stimulation and response even 1 year after vaccination. Moreover, the findings suggest that there are differences in immune responses and inflammatory cytokine levels based on gender and age. Additionally, this study observed that the interval following vaccination affects cytokine levels. These observations may be linked to the persistent production of spike protein and highly inflammatory nature of mRNA–lipid nanoparticle (LNP). The potential risks associated with a mRNA vaccine that prompts human cells to become targets for autoimmune responses cannot be thoroughly evaluated without precise knowledge of the distribution and behavior of LNPs and mRNA, along with the spike protein production dynamics.

The specific cytokine response following COVID‐19 vaccination can vary depending on factors such as the type of vaccine, individual immune system characteristics, and any pre‐existing conditions. All the subjects participated in this study took Pfizer vaccine and experienced significant increase in serum cytokine, chemokine, and growth factor levels including FGF2, IFNγ, IL1β, IL4, IL6, IL7, IL17E, MCP1, MCP3, TNFα, and VEGFA. These cytokines play various roles in the immune response and inflammation associated with COVID‐19. Studies have shown that the cytokine triad of IL1β, IL6, and TNFα is associated with post‐acute sequelae of COVID‐19 [22]. Additionally, chemokines produced by monocytes and macrophages, such as MCP1, MCP3, and IFNγ are directly related to the survival of COVID‐19 [23]. Furthermore, cytokines like IL4, IL6, and IL7 have been identified as part of the human high sensitivity cytokine panel and are associated with immune responses [24]. IL6 is seen as an important cytokine in the development of an antigen‐specific humoral response during certain infections [25]. The increase of IL6 can be associated with the onset of autoimmunity and autoinflammatory reactions [26] and this is consistent with the mechanism of autoimmune inflammatory reactions observed as an adverse effect upon vaccination [5, 27]. VEGFA and FGF2 are involved in the immune response and can be activated by the S protein of the SARS‐CoV‐2 [28]. The release of VEGFA and FGF2 may contribute to the regulation of immune responses and the formation of new blood vessels in response to vaccination [28]. Another study mentions that the concentrations of IL1, IL6, and TNF increased significantly on day 3 after the first dose of the ChAdOx1 nCoV‐19 vaccine [29]. Increased inflammatory response following the initial dose of the SARS‐CoV‐2 vaccine are probably linked to the inflammatory characteristics of LNPs. LNPs trigger various inflammatory pathways, resulting in the release of cytokines like IL1β and IL6, which can both initiate and perpetuate local and systemic inflammation [30]. This suggests that the vaccine can induce an immune response that involves the production of these cytokines.

Sexual dimorphism in cytokine expression was observed in the present study, supporting evidence specific cytokine profile and their levels can vary among individuals based on different characteristics including sex [7]. Sex hormones, such as testosterone and estrogen, play diverse roles in immune responses. These hormones can influence physiological functions and have effects on the regulation of immune functions. Testosterone is generally considered to be immunosuppressive, while estrogen, the female sex hormone, has different effects on immune responses [31]. Estrogen receptors are expressed in various immune cells, indicating their involvement in immune regulation [32]. Estrogen is recognized for its capacity to reduce the production of T and B cells, improve the functioning of B cells, and impact the development of T cells. Furthermore, estrogen plays a regulatory role in multiple cytokines, such as IL1, IL10, and IFNγ, which are involved in regulating the immune response. While estrogen can stimulate the immune system, progesterone and androgens act as hormones that suppress the immune system, counteracting the pathways influenced by estrogen [32, 33]. Results of the current study revealed that in comparison to women, men experienced more significant increase in pro inflammatory cytokines including IL4, IL6, and TNFα. These results are contrary to the findings reported in the literature where women tend to exhibit greater inflammatory, antiviral, and humoral immune responses compared to males, potentially due to the influence of sex hormones such as estrogen and testosterone. Research suggests that androgens, including testosterone, also have immune‐suppressive effects on the immune response [32, 34]. In comparison to estrogen, testosterone may predispose men to a more widespread COVID‐19 infection. Low serum levels of testosterone, which are often observed in seriously ill individuals, especially elderly men, may contribute to a poor prognosis or increased risk of death [32, 34]. Previous research has indicated variations in vaccine response based on sex, with females exhibiting higher vaccine efficacy but more severe adverse reactions compared to males across various vaccines such as those for influenza, hepatitis B, and yellow fever [35, 36, 37, 38]. This difference in response may be attributed to a greater number of B cells leading to increased antibody production in females, as well as heightened immune cell activation by female sex hormones (estrogen and progesterone) and suppression by male sex hormones (testosterone) [39, 40].

As people age, their immune system undergoes changes that can affect their response to vaccines, including the COVID‐19 vaccine. Aging can lead to a decline in immune function, a phenomenon known as immunosenescence [35]. This can result in a reduced ability to mount a robust immune response to vaccination, leading to poorer vaccine efficacy in older individuals. Several factors contribute to the poor vaccine response in aging individuals. One factor is the decline in the production of new immune cells, such as T cells and B cells, which are crucial for mounting an effective immune response Additionally, aging can lead to changes in the composition and function of immune cells, impairing their ability to recognize and respond to pathogens. Results of the current studies shown that in comparison to adolescents, older adults experienced more pronounced increase in EGF, IL6, MCP1, and TNFα while VEGFA decreased. The poor vaccine response in older individuals has been observed not only with the COVID‐19 vaccine but also with other vaccines, such as the influenza vaccine. Studies have shown that older adults may have a lower frequency of specific immune cells, such as Spike‐specific CD4^+^ and CD8^+^ T cells, in response to COVID‐19 mRNA vaccination [35, 41, 42, 43]. These age‐related changes in the immune system can contribute to reduced vaccine efficacy and potentially increase the risk of severe outcomes from infections like COVID‐19. The impact of immunosenescence on the diminution in vaccine effectiveness has been observed with other vaccines like influenza, varicella zoster, and the combined vaccine for tetanus, diphtheria, and pertussis [35, 41, 42, 43].

Research suggests that cytokines may persist for months after COVID‐19 vaccination [44, 45, 46, 47]. While the exact duration and implications of cytokine persistence are still being studied, several studies have indicated prolonged cytokine responses following vaccination. One study found that cytokine production may persist for months after vaccination, suggesting that the immune response continues beyond the acute phase [44]. Another study observed sustained decreases in cytokine responses to viral stimuli 6 months after COVID‐19 vaccination [45]. In the present study, significant increase in cytokines, chemokines and growth factors including FGF2, IFNγ, IL1β, IL4, IL6, IL7, IL17E, MCP1, MCP3, TNFα, and VEGFA were observed in participants who took their last dose 4 months prior or less. However, in participants who took their last vaccine dose before 5 months or more, only TNF, IL4, MCP3, and VEGFA concentrations were observed to be significantly elevated. However, these differences vanished when the results were adjusted for age differences. Previous studies have shown that systemic inflammation can gradually subside after vaccination. For example, a case report on COVID‐19 vaccination mentioned that systemic inflammation gradually decreased after the use of anti‐inflammatory drugs and steroids [46]. Another study reported reduced inflammation in cytokine/chemokine levels after vaccination [47]. Rubin et al. [48] compared vaccinated participants with recovered COVID‐19 patients and reported that COVID‐19 patients had higher cytokine levels but lower antibody levels than vaccinated participants. They found that serum levels of IL6, TNFα, IL8, vascular cell adhesion molecule‐1, and matrix metalloproteinase‐7 were lower in the vaccinated group than the recovered group. Zhu et al. [49] further demonstrated that fully vaccinated participants showed significantly lower levels of inflammatory markers both at the onset and during recovery from symptomatic COVID‐19 than unvaccinated participants, and concluded that vaccination correlated with a reduction in inflammation, both in the short term and over an extended period. However, the absence of unvaccinated participants or those with prior COVID‐19 infection in the present study, limits the ability to directly align its findings with those reported by Rubin et al. [48] and Zhu et al. [49].

Polykretis et al. [27] provided strong histological evidence indicating that genetic vaccines exhibit off‐target biodistribution, leading to the synthesis of spike protein and potentially triggering autoimmune‐inflammatory reactions. These effects are detectable even in terminally differentiated tissues, which may result in clinically observable pathological damage [50, 51, 52, 53]. Baumeier et al. [50] also identified vaccine‐derived spike protein in cardiomyocytes through immunohistochemical analysis in 9 out of 15 individuals with postvaccination myocardial inflammation, all of whom were negative for active SARS‐CoV‐2 infection. This finding suggests localized synthesis of the viral protein in cardiac tissue and implies an autoimmune mechanism linked to vaccination [50]. Mörz [51] documented the expression of vaccine‐encoded spike protein in the cerebral and myocardial tissues of a patient who developed multifocal necrotizing encephalitis following BNT162b2 administration. Additionally, the association between COVID‐19 vaccination and severe cardiovascular adverse events, particularly among younger, otherwise healthy individuals, has gained increasing recognition in the scientific literature [27, 54, 55, 56]. Postmortem analyses have further confirmed vaccine‐induced pathologies as direct causes of mortality in certain cases [52, 53, 57, 58]. Polykretis et al. [27] emphasized the necessity of conducting biodistribution studies for COVID‐19 genetic vaccines and advocated for age‐specific harm‐benefit evaluations to inform vaccination strategies.

This study has few limitations. First, the results are based on young adults and so the findings cannot be generalized to elderly participants. Information on participants who had breakthrough infections were not taken into consideration, and this may affect the cytokine levels independent from mRNA vaccines. Furthermore, the effects were limited COVID‐19 vaccines of the mRNA type and may not necessarily be true for other types such as viral vectors like ChAdOx1‐S (AstraZeneca). Lastly, this study does not account for the variability in individual immune responses and lifestyle factors that may influence inflammatory markers. Factors such as age, pre‐existing health conditions, diet, and physical activity levels can all play a significant role in the inflammatory response to mRNA vaccines. Without controlling for these variables, it becomes challenging to draw definitive conclusions about the direct impact of mRNA vaccines on inflammatory factors. Future research should aim to include a broader range of individual characteristics to provide a more comprehensive understanding of how these factors interact with vaccine efficacy.

## 5 Conclusions

In conclusion, this longitudinal study assessed the impact of mRNA COVID‐19 vaccine in adults who receive three doses on cytokine profile. The finding of this study showed that vaccination led to elevated cytokine levels, indicating ongoing stimulation and response of the immune system even a year postvaccination. These results may be associated to the persistent production of spike protein and highly inflammatory nature of mRNA‐LNP. The findings also revealed that males and adults experienced surge in pro‐inflammatory cytokines compared to females and adolescents. The recognition of distinct adverse symptoms associated with gender and age provides an opportunity to enhance comprehension of immunological variances related to sex and age, and to create vaccines that are safer and more efficient.

## Author Contributions

**Amani Alghamdi:** conceptualization, funding acquisition, writing – review and editing. **Syed Danish Hussain:** formal analysis, writing – original draft. **Kaiser Wani:** investigation, validation, methodology, writing – review and editing. **Shaun Sabico:** writing – review and editing, supervision, investigation. **Abdullah M. Alnaami:** investigation, methodology, writing – review and editing. **Osama Emam Amer:** investigation, methodology, writing – review and editing. **Nasser M. Al‐Daghri:** supervision, writing – review and editing, conceptualization.

## Ethics Statement

The research was carried out in accordance with the principles of the Declaration of Helsinki and approved by the King Saud University College of Medicine's ethics committee on February 16, 2023 (approval no. 23/0054/IRB‐A).

## Consent

Informed consent was obtained from all the subjects involved in the study.

## Conflicts of Interest

The authors declare no conflicts of interest.

## Supporting information

STROBE_Checklist.

## Data Availability

The data that support the findings of this study are available from the corresponding author upon reasonable request. The corresponding author may provide the data described in this study upon request.
